# Off‐label and unlicensed medicines to hospitalised children in Norway

**DOI:** 10.1111/jphp.12581

**Published:** 2016-06-23

**Authors:** Arna Teigen, Siri Wang, Bich Thuy Truong, Kathrin Bjerknes

**Affiliations:** ^1^ Hospital Pharmacy EnterprisesSouth Eastern NorwayOsloNorway; ^2^ School of PharmacyUniversity of OsloOsloNorway; ^3^ Department of Medicinal Product AssessmentNorwegian Medicines AgencyOsloNorway

**Keywords:** hospitalised children, off‐label, paediatric, unlicensed medicines

## Abstract

**Objectives:**

The aim of this study was to investigate the use of off‐label (OL) and unlicensed (UL) medicines to hospitalised children in Norway, to add to the current knowledge on use of medicines in this vulnerable patient group.

**Methods:**

The study was performed as a cross‐sectional prospective study. Medication was classified as on‐ or off‐label based on the comparison with the SmPC regarding age, indication, dosage, route of administration and handling of the product. UL products were classified as imported or pharmacy produced.

**Key findings:**

More than 90% of children receiving medicines in our study were given OL or UL medicines. More patients received OL (83%) than UL (59%). Route of administration was the most frequently observed OL category. The vast majority of the OL prescriptions were for ‘off‐patent’ products. One‐third of products prescribed were UL.

**Conclusions:**

The study confirms that medicines to children in hospital to a significant degree are being used outside or without authorisation, in spite of recent paediatric regulatory initiatives. More data are still needed on efficacy and safety of medicines used in children, data to be incorporated in the SmPC. In addition, suitable formulations are needed to ensure optimal dosing and adherence without risky manipulations.

## Introduction

Authorisation of a medicinal product for a specific use intends to ensure sufficient quality, efficacy, and patient safety. Such ‘labelling’ is in general considered the gold standard for optimal use of medicinal products. The professional decision‐making by the treating physician should always rely on best available evidence. Proper authorisation of a medicinal product for paediatric use is a vital tool in this regard. Nevertheless, for use in children it is well known that most medicinal products are neither designed, documented, nor authorised for this patient group and therefore have to be used outside this ‘label’.^1^, ^2^ Prospective studies from Europe have shown increased risk of adverse drug reactions when children are using OL or UL medicines,^3^, ^4^, ^5^, ^6^ underpinning the need for appropriately authorised medicines also for this age group. However, OL use of medicines might also be an important instrument to optimise treatment in children and do not per se imply improper or illegal use. The challenges of such OL practice may include insufficient information on dosing, efficacy and safety. In addition, inappropriate formulations may affect the actual dose and the need for manipulation and could have impact on safety as well as adherence of treatment. Alternatively, products might be imported or compounded by the pharmacy; however, use of such UL preparations may have other challenges such as unpredictable availability, variable quality, lack of suitable product information and high price.

Through the last 20 years, there has been an increased awareness about the lack of medicines documented for use in children. Regulatory actions have been taken worldwide to improve the situation by posing requirements to the pharmaceutical industry to develop new relevant products also for children.^7^, ^8^ In addition, work is ongoing to share the existing knowledge on authorised substances^9^, ^10^ and to push for transparency on ongoing clinical studies.^11^

In spite of the overall agreement regarding the importance of these initiatives, this work is a test of patience, and progress may seem slow.^8^, ^12^, ^13^ It is therefore important to keep on monitoring the use of medicinal products in children both locally and globally. The current study is a part of this international work to focus on the improvement in both clinical practice and the availability of drugs for children and is the first study on OL and UL drug use in hospitalised children in Norway.

## Methods

The study was performed as a cross‐sectional prospective study at two hospitals in Norway, the Oslo University hospital, Ullevål, and Akershus University hospital. One neonatal and three paediatric wards with 67 beds in total were included, covering mainly gastrointestinal disorders, endocrinology, neurology, respiratory diseases and infections in patients 0–17 years. The study period at Oslo University hospital was September–October 2013 and at Akershus University hospital September–December 2014. All patients hospitalised during the study periods were evaluated for potential inclusion. Patients were eligible for inclusion if using medicines during the hospital stay, excluding standard intravenous fluids, heparin, blood products, total parenteral nutrition products, creams and ointments. Inclusion was confirmed after consent by the patient or a guardian.

Medication charts were checked regularly during the study period, starting within the first day of the hospital stay or the first day of the data collection period for patients who were already admitted. All medications were registered for the entire hospital stay within the study period. For each patient, the age and weight were recorded. In addition, gestational age was recorded for patients in the neonatal ward. For each drug prescribed, the specific route of administration, dosing and the indication for use were recorded. Additional information regarding indication was provided from the prescribing physician when needed. Information regarding the practical handling of the drug was obtained from the nurse responsible for the patient, or from guardians where relevant, as this information was often lacking in the medical records. Descriptive analyses were performed on the data structured using Microsoft Office Excel.

The following categories of medicinal products were not recorded: standard intravenous fluids, heparin, blood products, total parenteral nutrition products, creams and ointments. Medicines prescribed to be given ‘on demand’ were only evaluated if actually given to the patient.

Products were classified as licensed if marketing authorisation was granted in Norway. The actual use of a licensed medicine was compared to the Summary of Product Characteristics (SmPC) for five categories: age, indication, dosing, route of administration and practical handling of the drug. The following SmPC sections were evaluated: 4.1 (indication), 4.2 (posology and administration), 4.3 (contraindications), 6.2 (incompatibilities) and 6.6 (special precautions for disposal or other handling). Each prescription was classified as on‐ or off‐label and could be OL for more than one category. If indication or age was considered OL, dosing was not assessed.

When exact age or weight were not specified in the SmPC, the following terminology was applied: preterm born <32 weeks gestational age, neonates ≤28 days, infants ≤1 year, young children 1–4 years, children ≤12 years, adolescents 13–17 years and adults ≥18 years. For indication, focus was on the main condition and not the exact SmPC wording. Dosing was assessed as OL if deviation was observed in either dosing frequency or single or maximal daily dosing. UL medicines were classified as pharmacy produced or imported and were not further evaluated. Two pharmacists independently performed the classification of prescriptions and products as on‐label, off‐label or unlicensed. Regional ethics committee for medical and health research ethics found no ethical approval necessary (2013/588). The Data Protection Officer for Research at the two hospitals approved the study in advance.

## Results

Of the 400 hospitalised patients at the wards in the study periods, 179 were included in the study. Main causes for not including the remaining 221 patients were no (or only excluded) medicines prescribed (41%) or consent not obtained (59%). The age distribution in the study population is outlined in Table 1. Approximately 60% of the patients were below 2 years. A total of 205 different medicinal products were administered to the study population during the study (Table 3). Patients received on average 5.2 prescriptions. Most of the patients (91%) received OL or UL medicines (Table 1). More patients received OL compared with UL (83% vs 59%) (Table 1).

**Table 1:** Patients receiving off‐label (OL) or unlicensed (UL) medicines

Age group	Total number of patients (*n*)	Patients receiving OL *n* (%)^a^	Patients receiving UL *n* (%)^a^	Patients receiving OL or UL *n* (%)^a^
0–28 days	50	43 (86)	32 (64)	49 (98)
Of these				
Preterm	25	20 (80)	18 (72)	25 (100)
Term	25	23 (92)	14 (56)	24 (96)
28 days–23 months	55	45 (82)	36 (65)	51 (93)
2–5 years	22	20 (91)	18 (82)	22 (100)
6–11 years	28	23 (82)	13 (46)	24 (86)
12–17 years	24	17 (71)	7 (29)	17 (71)
Total	179	148 (83)	106 (59)	163 (91)

a Percentage of total number of patients in the age group.

### Off‐label

OL use of medicines in the study group was frequent for all age groups as outlined in Table 1. In all age groups, most patients received OL medicines (71–92%) and almost half of the prescriptions were OL (40–47%) (Tables 1 and 2).

**Table 2:** Off‐label (OL) or unlicensed (UL) prescriptions in the study group

Age group	Number of prescriptions (*n*)	OL prescriptions *n* (%)^a^	UL prescriptions *n* (%)^a^	OL or UL prescriptions *n* (%)^a^
0–28 days	267	113 (42)	100 (37)	213 (80)
Of these				
Preterm	166	67 (40)	77 (46)	144 (87)
Term	101	46 (46)	23 (23)	69 (68)
28 days–23 months	275	118 (43)	70 (25)	188 (68)
2–5 years	130	60 (46)	35 (27)	95 (73)
6–11 years	137	65 (47)	22 (16)	87 (64)
12–17 years	121	54 (45)	13 (11)	67 (55)
Total	930	410 (44)	240 (26)	650 (70)

a Percentage of total prescriptions in the age group.

**Figure 1:**
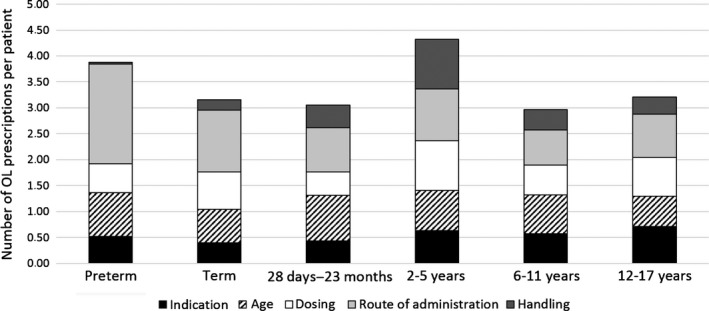
Number of off‐label (OL) prescriptions per patient in the OL categories.

The frequencies of the OL categories recorded regardless of age group were as follows: route of administration (31%), age (23%), dosing (19%), indication (16%) and handling (11%). No single category predominated. Distribution of the different categories within each age group is outlined in Figure 1. Overall, when correcting for number of patients per age group, similar frequency of categories was observed in all age groups except for handling of the drug. OL handling was most frequently seen in the age group of 2–5 years, and rarely seen in the preterm group. Route of administration was the most common OL category in the premature group; 72% of 67 OL prescriptions were attributed to this group.

**Figure 2:**
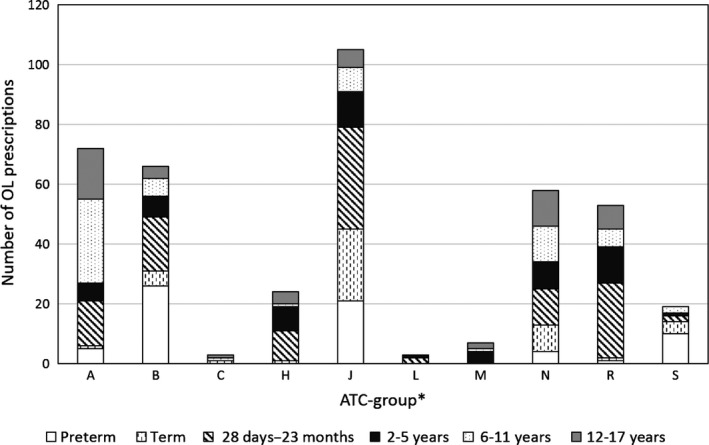
Number of off‐label (OL) prescriptions registered by ATC in the age groups. ATC*, Anatomical Therapeutic Chemical; A, alimentary tract and metabolism; B, blood and blood‐forming organs; C, cardiovascular system; H, systemic hormonal preparations; J, anti‐infectives for systemic use; L, antineoplastic and immunomodulating agents; M, musculo‐skeletal system; N, nervous system; R, respiratory system; S, sensory organ.

OL prescriptions by ATC groups are outlined in Figure 2. The medicines prescribed OL are spread on multiple ATC groups potentially representing a variety of indications. However, it also reflects the study patient population; for example, few products were prescribed OL in the cardiovascular group (C) and the antineoplastic group (L) and these groups of patients were under‐represented in our study material.

**Figure 3:**
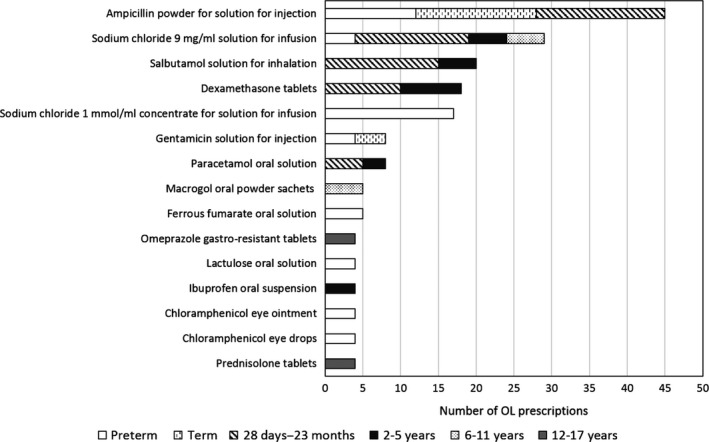
The most frequently prescribed off‐label (OL) medicines.

The most frequently prescribed OL medicines are shown in Figure 3. Only a few products were used OL consistently over most age groups, as, for example, sodium chloride 9 mg/ml solution for infusion was prescribed for inhalation to almost all age groups, but is only authorised for parenteral use, and route of administration was therefore assessed as OL. However, most products had a more narrow OL age range and were often prescribed OL in only one age group. Ampicillin powder for injection or infusion is used intravenously in all age groups but is only authorised as intramuscular administration for children under 40 kg; therefore, route of administration was assessed as OL for the youngest age groups. All of the most frequently prescribed OL medicines were older products, no longer covered by patent or protection.

### Unlicensed

Approximately one‐third (*n* = 61) of the prescribed products in the study group were UL (Table 3). All age groups received UL medicines, with highest proportion in the age group of 2–5 years (82%) and lowest in adolescents (29%) as outlined in Table 1. Percentage of prescriptions of UL medicine varied between the age groups (11–46%) (Table 2).

**Table 3:** Number of unlicensed (UL) medicinal products administered

Age group	Total medicinal product (*n*)	Unlicensed drug *n* (%)^a^	Imported (*n*)	Pharmacy produced (*n*)
0–28 days	63	18 (29)	10	8
Of these				
Preterm	35	12 (34)	8	4
Term	28	6 (21)	2	4
28 days–23 months	88	29 (33)	18	11
2–5 years	70	19 (27)	14	5
6–11 years	76	15 (20)	13	2
12–17 years	73	9 (12)	6	3
Total number of products	205	61 (30)	41	20

a Percentage given as part of total medicinal product within the age group.

Table 3 shows the number of UL products prescribed per age group, split into imported products and products made by the hospital pharmacy. Overall, the ratio of imported vs pharmacy produced was about 2 : 1, showing that commercial alternatives are often available outside Norway.

The most frequently prescribed (≥3) UL medicines in the different age groups are listed in Table 4. Overall, racemic adrenaline solution for inhalation (30), caffeine oral solution (27), ibuprofen suppositories (18) and vitamin K oral drops (14) were the most frequently prescribed medicines in the study period regardless of age group. Apart from a few products being used in a broad range of ages (racemic adrenaline, ibuprofen), most products had age‐specific use; for example, caffeine oral solution and vitamin K oral drops were only prescribed to preterm neonates, betamethasone‐soluble tablets only to the age range 28 days–23 months and morphine solution for injection only to term neonates. The medicines belonged to a range of ATC groups, with group N (nervous system) and A (alimentary tract and metabolism) most frequently represented.

**Table 4:** The most frequently prescribed unlicensed (UL) medicines

Age	UL product
0–27 days	
Preterm	Caffeine oral solution
	Vitamin E oral solution
	Probiotic capsules (lactobacillus acidophilus, bifidobacterium bifidum)
	Vitamin K oral drops
	Multivitamin oral drops
	Folic acid oral solution
Term	Morphine 1 mg/ml solution for injection
	Vitamin K oral drops
28 days–23 months	Ibuprofen suppositories
	Racemic adrenaline solution for inhalation
	Betamethasone‐soluble tablets
	Gentamicin 20 mg/2 ml solution for injection
	Codeine oral liquid
2–5 years	Racemic adrenaline solution for inhalation
	Ibuprofen suppositories
	Codeine oral solution
6–11 years	Oil–glycerol enema
	Amoxicillin with clavulanic acid oral suspension
12–17 years	Ibuprofen suppositories

## Discussion

The present study showed that 91% of the included paediatric patients received at least one off‐label or unlicensed (OLUL) drug prescription. The majority (70%) of the prescriptions was OLUL. Previous studies from other European countries report patients receiving OLUL medicines in the range 42–100%, 16–87% of the prescriptions being OLUL, as summarised by Magalhaes.^14^ Recent studies are in line with these findings.^15^, ^16^ The definition of OL and UL is not similar for all studies, and this might partly explain the variations reported. In addition, in our study, only patients receiving medicines during the hospital stay were included, and the results are reported on this population. The different approaches in defining the study population might not always be comparable.

Similar to other studies, the neonates remains to be one of the age groups where OLUL prescribing is seen most often;^14^, ^17^ in our study population, all the preterm infants received one or more OLUL prescription. The fact that no patients in the study group received only licensed and on‐label prescriptions may not be surprising in the preterm group, but was also seen in the age group of 2–5 years. In general, the high proportions of patients receiving OLUL in this study is probably due to commonly used medicines (e.g. ampicillin, caffeine, racemic adrenaline) or scenarios (e.g. administration through enteral feeding tube) that were considered OLUL in the different age groups. As expected, fewer patients in the older age groups received OLUL medicines.

Interestingly, the frequency of OL prescriptions was similar for all age groups (40–47%), which was unexpected. We would have anticipated an increased rate of OL prescriptions in the younger cohorts as generally a product is more likely to be authorised in older age groups, more comparable to the adult cohort, have formulation suitable for the older age groups, etc. The same pattern, no clear age‐dependent OL prescription frequency, is, however, also observed in previous reports.^16^, ^18^

Route of administration was the most frequent OL category, and this is in contrast to most other studies, where dose, age and indication are more frequently found.^14^ In one study where route of administration was the most frequently reported category,^16^ buccal administration of sedatives and analgesics at the surgical ward was the main explanation. In our study, administration via enteral feeding tubes contributed significantly to the OL category route of administration. As for all other categories, administration via enteral feeding tubes was considered OL if not described in the SmPC. It is unclear whether in other studies this has been handled similarly due to lack of details in definition of the categories; nevertheless, this scenario was frequently observed in our data, and rarely described in the SmPC. This element is one factor potentially explaining the high overall fraction of patients receiving OLUL medicines in our study.

Splitting of tablets or dispersing into small volumes were the most typical OL handling. The low incidence of OL handling in neonates reflected the higher frequency of UL liquid medicines in this age group, or an overall limited number of products used. In theory, different handling procedures for each prescription could have happened without the notice of the study pharmacists; some handling considered ‘on‐label’ at the time of recording could occasionally have been handled off‐label. The numbers on prescriptions with OL handling might therefore be slightly underestimated.

We noted significant differences in the level of details of the SmPCs, newer products often having more detailed SmPC compared with older products; for example, the assessment of OL indication for newer products might require a level of details in the actual patient indication that is not feasible to obtain in this type of study. For some products, no age information was given in the SmPC, and thus, the use of the product was considered OL for all paediatric age groups. In other studies, this scenario may have been categorised as ‘lack of paediatric licence/information’.^14^

Prescriptions were often classified as OL in more than one category. As mentioned above, administration via enteral feeding tube, the main reason for OL route of administration, also often implied OL handling like crushing of tablets, opening capsules and dispersing into liquid before administration. Similarly, a product not being authorised in a particular age group (OL age) would often imply OL handling. This potential covariation between OL categories may partly explain the even distribution between OL categories.

One‐third of the products prescribed to children in this study were not licensed in Norway. Being a small country in the European setting, the number of licensed paediatric products is limited, as pharmaceutical companies tend to restrict the number of products actually put on the market if the revenue is sparse. Two‐third of the UL medicines in this study were imported, confirming that several commercial products are available in Europe but not marketed in Norway. The most frequently prescribed UL medicines were racemic adrenaline solution for inhalation, caffeine oral solution, ibuprofen suppositories and vitamin K oral drops. Racemic adrenaline has been imported and used extensively until a recent study documented the lack of efficacy compared with saline in the treatment of bronchiolitis.^19^ A caffeine product (Peyona^®^, Chiesi Farmaceutici, Parma, Italy) was recently authorised in Norway, and a future reduction in use of UL caffeine is thus expected.

Most of the UL products contained active pharmaceutical ingredients for which no authorised products were available in Norway, for example caffeine and betamethasone. Only a few hospital preparations were seen when commercial products were available in other countries, which is in line with a national policy to import medicines with documentation approved by a national authority rather than produce locally.

The major limitations in our study relates to the limited size and population. Covering only one neonatal and three paediatric wards at two hospitals, the generalisability of the data is limited although it gives an indication of the OLUL use at two of the largest university hospitals in Norway. Being a cross‐sectional study, the time span is limited. Previously reported therapeutic areas of frequent OLUL use like cardiology^14^, ^20^, ^21^ and oncology^22^, ^23^ were not well represented. However, the areas where more products are authorised for children, like anti‐infectives, anti‐asthmatics and analgesics, are included in the study population, and thus, the overall picture of OLUL use is most likely not overestimated.

The apparently slow progress in increasing information and availability of medicines for children might have several explanations. First, there is a significant lag time from regulatory initiatives are put in place until data and products are available. More important probably, the majority of products used in children are off‐patent, and therefore, many regulatory incentives have limited applications. Interestingly, in our study, all of the fifteen most frequently prescribed OL medicines were from old, well known substances and products no longer patented. More focus needs to be put on how to enforce collection of new and existing data for such products, as well as on the availability of appropriate formulations for these old substances.

## Conclusion

This study confirms that the use of medicines outside or without authorisation when treating hospitalised children is still at a significant level. This despite the initiatives taken by the European authorities to develop more medicinal products documented for children. More than 90% of the paediatric patients studied received medicines that were either not authorised in Norway or given outside authorisation with respect to age group, indication, dosing, route of administration or practical handling of the drug. While waiting for improved availability of appropriately authorised products, significant attention should be put on ensuring that tools and systems are available to best possibly inform prescribers on appropriate use of medicines for children. This is particularly important for old products where regulatory initiatives are likely to have less impact, but which are of paramount importance for children.
